# Application of contrast-enhanced ultrasonography for large cell neuroendocrine carcinoma in the urinary bladder: a case report

**DOI:** 10.1186/s12880-020-00447-6

**Published:** 2020-05-03

**Authors:** Wei Li, Ze-Zhen Su, Ji-Hui Kang, Xiao-Yan Xie, Xiao-Hua Xie, Bo-Wen Zhuang

**Affiliations:** 1 Department of Medical Ultrasonics, Institute of Diagnostic and Interventional Ultrasound, The First Affiliated Hospital of Sun Yat-Sen University, 7/F, 2nd Building, 58# Zhongshan Road 2, Guangzhou, 510080 People’s Republic of China; 2 Department of Pathology, The First Affiliated Hospital of Sun Yat-Sen University, NO.58 Zhongshan Road 2, Guangzhou, 510080 People’s Republic of China

**Keywords:** Bladder neoplasm, Large cell neuroendocrine carcinoma, Contrast-enhanced ultrasound, Ultrasound

## Abstract

**Background:**

Large cell neuroendocrine carcinoma (LCNEC) of the urinary bladder is an uncommon malignant bladder tumor, and the overall prognosis is poor. Contrast-enhanced ultrasound (CEUS) provides a new effective modality for tumor detection and diagnosis.

**Case presentation:**

A 30-year-old man complained of repeated painless gross haematuria for half a month. Conventional ultrasound demonstrated a hypoechoic solitary lesion with hyperechoic margins measuring 3.4 × 3.1 cm in the anterior wall of the bladder. Superb microvascular imaging (SMI) showed a strong flow signal in the mass. CEUS revealed that the lesion was characterized by hyper-enhancement in the early phase and hypo-enhancement in the late phase. The entire bladder wall was disrupted by homogeneous hyper-enhanced tumor tissue on CEUS. Time-intensity curves (TICs) showed a rapid wash-in with a high maximum signal intensity (SI) and quick wash-out. Finally, partial cystectomy was performed and the pathological examination confirmed the diagnosis of LCNEC with invasion into the whole layer of the bladder wall.

**Conclusion:**

This case suggested that CEUS was a valuable imaging method to detect and diagnose LCNEC in the bladder, and that CEUS can provide information related to the depth of wall invasion and the microvasculature.

## Background

Neuroendocrine tumors are most common in the respiratory and gastrointestinal tracts but constitute only approximately 1% of bladder tumors [1, 2]. Most of neuroendocrine carcinomas in the urinary bladder is represented by small cell neuroendocrine carcinoma while large cell neuroendocrine carcinomas (LCNECs) are extremely rare [2]. LCNEC of the urinary bladder was first reported in 1986 [3], and since then, fewer than 30 cases have been reported in the literature. LCNEC of the urinary bladder is characterized by poor differentiation and strong invasiveness and is frequently detected at an advanced stage when initially diagnosed, leading to high metastatic potential and poor prognosis [4]. The absence of specific clinical symptoms and laboratory findings combined with high mortality make detection and diagnosis through imaging studies extremely crucial. The various appearances of LCNCB on computed tomography (CT) images have been depicted in case reports and in a few small series [5, 6]. However, the ultrasound (US) appearances of this tumor, particularly on contrast-enhanced ultrasound (CEUS), has not been clearly described. CEUS can provide real-time visualization of contrast-enhanced patterns, which is useful for the differential diagnosis of urinary bladder lesions [7–9]. In addition, using the pattern of time-intensity curves (TIC) can reflect the tumor microvessel density, which may be helpful in evaluating tumoral neovascularisation in bladder tumors [8, 10].

In this manuscript, we report a case of LCNEC in the urinary bladder imaged by conventional US and CEUS. To the best of our knowledge, this is the first report on CEUS manifestations of LCNEC.

## Case presentation

A 30-year-old man presented with recurring painless gross haematuria for half a month but without urinary tract infections or lower back pain. He had an unremarkable history of cigarette smoking, clinical history, and physical examination. On admission, his laboratory results showed routine urinalysis with elevated numbers of red blood cell count and leucocytes. Furthermore, tumor marker levels were within their normal ranges.

**Fig. 1:**
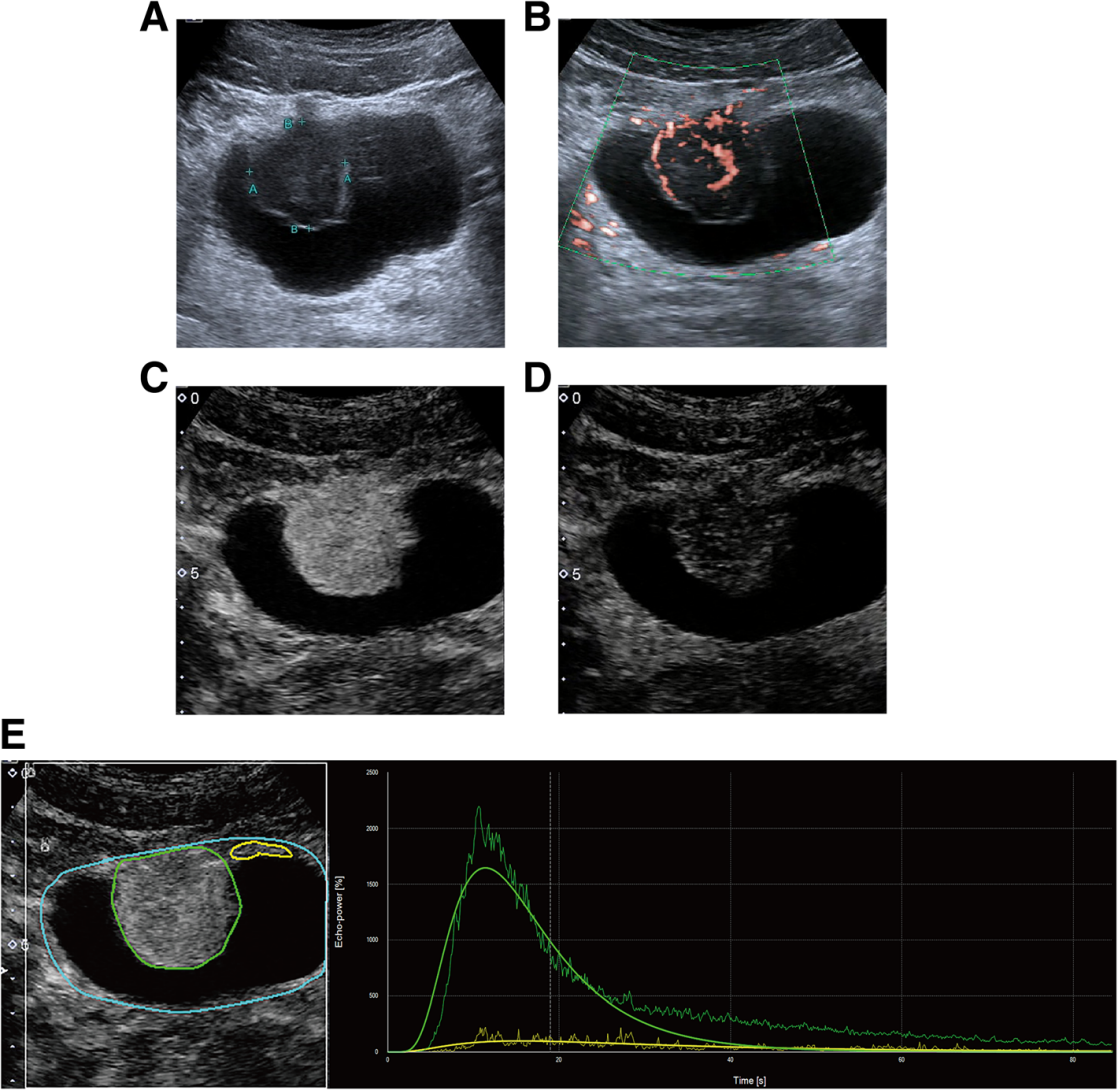
Ultrasonography and contrast-enhanced ultrasound (CEUS) imaging of large cell neuroendocrine carcinoma (LCNEC) in the urinary bladder. **a** Sonography showed a hypoechoic mass with hyperechoic margins in the anterior wall of the urinary bladder. **b** Superb microvascular imaging (SMI) showed strong blood flow signals in the mass. **c** CEUS imaging showed that the lesion achieved hyper-enhancement in the early phase in comparison to the bladder wall. **d** The lesion showed hypo-enhancement compared to the bladder wall. **e** Time-intensity curves (TICs) was created by analysing the ROI (green) positioned in the tumor and the reference ROI (yellow) in the bladder wall. TICs showed the lesion was initially enhanced at 9 s, the time to peak was 13 s and wash-out occurred at 40s

Ultrasonography was performed with an Aplio500 device (Toshiba Medical Systems, Tokyo, Japan) equipped with a 375BT convex transducer (frequency range 3.0–6.0 MHz). Conventional US revealed the presence of a large cauliflower shaped mass (3.4 × 3.1 cm), located at the anterior wall of the bladder, and the mass did not move with changes in body position. The mass exhibited uniform echogenicity, protruded into the lumen with hyperechoic margins and had a wide base (Fig. 1a). No invasion of the trigone of bladder and bilateral hydronephrosis was observed. Superb microvascular imaging (SMI) showed strong blood flow signals in the mass (Fig. 1b). CEUS was then performed with an injection of 2.4 ml US contrast agent (SonoVue, Bracco, Milan, Italy) followed by 5 ml 0.9% sterile saline flush through the antecubital vein. The examination was performed at a low mechanical index of 0.09. In the early phase, the lesion exhibited completely homogeneous enhancement that was obviously stronger than that of the normal bladder wall (Fig. 1c). At the same time, CEUS showed disruption of the entire bladder wall by homogeneous hyper-enhanced tumor tissue. Then, the lesion rapidly showed hypo-enhancement, resulting in a sharp contrast compared with the adjacent bladder wall (Fig. 1d). Next, we created TICs to exactly analyse the perfusion of the lesion. The lesion was initially enhanced on CEUS at 9 s compared to the bladder wall that was enhanced at 13 s. The tumor reached the peak enhancement at 13 s, and the strong enhancement was continuously maintained until 40 s. Subsequently, the microbubbles within the mass began to wash out, and the level of enhancement decreased to equivalent to that of the bladder wall (Fig. 1e). After 300 s, the microbubbles in the tumor were completely washed out. These CEUS features suggested a diagnosis of urinary bladder malignancy.

**Fig. 2:**
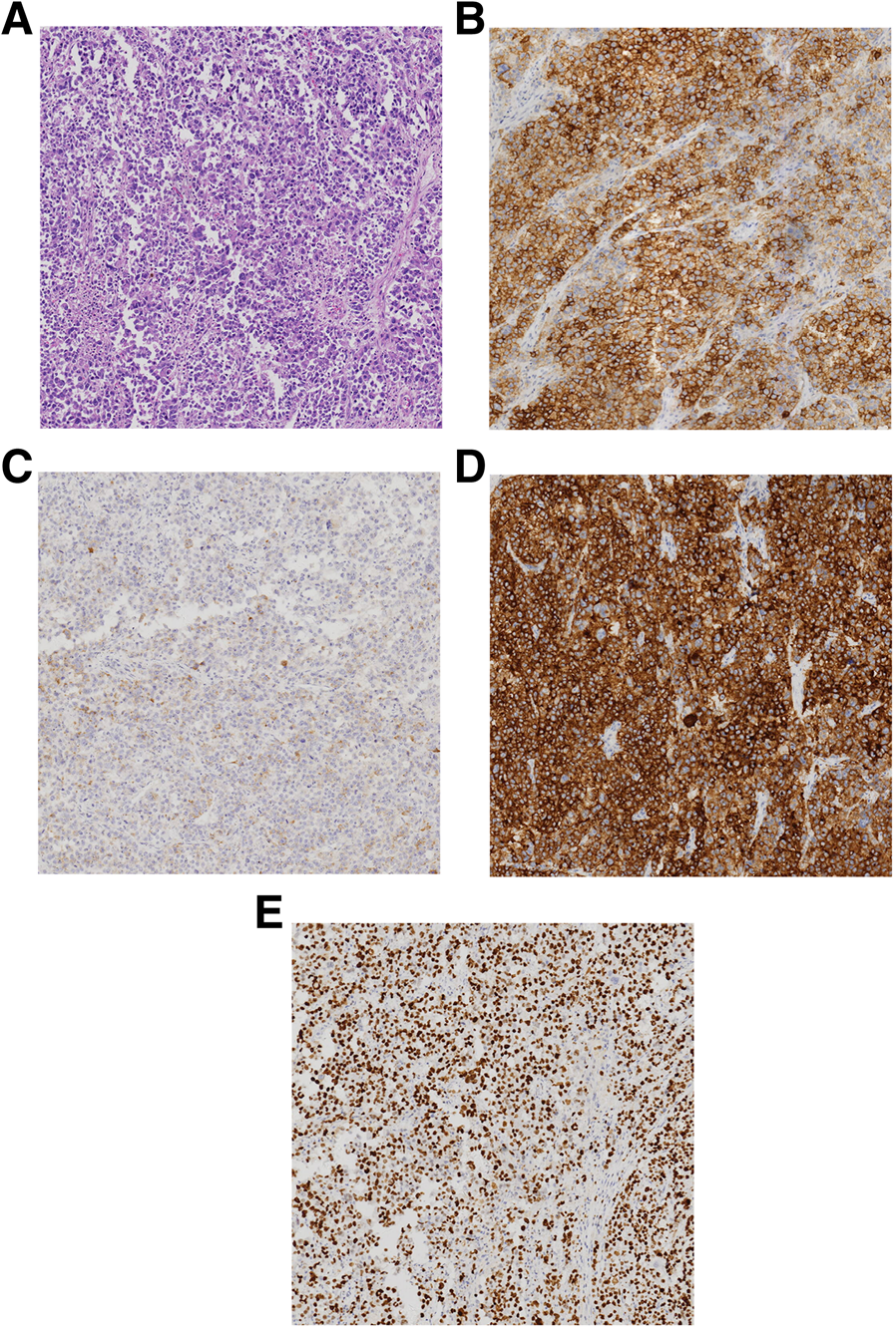
Histological findings of the mass. **a.** Haematoxylin and eosin staining revealed large tumor cells with polymorphic nuclei and organoids, trabecular growth, a coarse chromatin pattern, and prominent nucleoli. Immunohistochemical staining indicated that tumor cells were positive for CD56 (**b**), chromogranin A (**c**) and synaptophysin (**d**). The cellular proliferation marker Ki-67 was as high as 90% (**e**)

The patient underwent partial cystectomy under general anaesthesia without complications. Histological examination revealed large tumor cells with polymorphic nuclei and organoids, trabecular growth, a coarse chromatin pattern, and prominent nucleoli (Fig. 2a). Immunohistochemically, the neuroendocrine tumor differentiation markers CD56 (Fig. 2b), chromogranin A (Fig. 2c) and synaptophysin (Fig. 2d) were positive, suggesting neuroendocrine differentiation. The cellular proliferation marker Ki-67 was as high as 90% (Fig. 2e). Based on these findings, the tumor was diagnosed as LCNEC. The patient received six courses of postoperative adjuvant chemotherapy (cisplatin/etoposide) and he has now been free of recurrence for more than 2 years after surgery.

## Discussion and conclusions

LCNEC of the urinary bladder is an extremely rare malignant neoplasm with a high incidence of rapidly progressing and metastatic disease in clinical practice. This malignancy has a significant male sex predilection, as almost 80% of diagnosed subjects were men, while the mean age at the time of diagnosis is close to 60 years [1]. Smoking is the most important risk factor [11]. The most common clinical presentation is haematuria, which may be accompanied by flank pain, dysuria, ureteral obstruction and hydronephrosis [2]. The differential diagnosis includes urothelium carcinoma, lymphoma, small cell neuroendocrine carcinoma (SCNEC) or metastatic malignant round cell neoplasms.

Ultrasonography is usually the first imaging study performed and has been widely used for the identification of bladder lesions in clinical practice. However, the accuracy and sensitivity of US in the detection of bladder lesions is relatively low, and it is difficult to detect wall invasion and the microvasculature of the tumor [9, 12]. Various studies demonstrated that magnetic resonance imaging (MRI) and computed tomography (CT) using iodinated contrast media could be valuable for evaluating extravesical extension, tumoral infiltration and lymph node metastasis in bladder cancer [13]. However, CT and MRI scan are expensive and sometimes unsafe because of adverse reactions associated with using iodine as a contrast medium. In addition, some patients with high urea and creatine levels or in early stages of kidney disease may not be able to tolerate CT and MRI contrast medium and US may be useful in this scenario as well.

CEUS can play a significant role in the diagnosis of urinary bladder, and is helpful for evaluating the depth of wall invasion and detecting of microvasculature [9, 12]. Previous studies have explored the application of CEUS in the diagnosis and staging of bladder urothelium carcinoma, but there are no reports of the use of CEUS to diagnose LCNEC. Caruso et al. [7] reported that on CEUS, a tumor was considered superficial when the relatively hypo-enhanced muscle layer of the bladder wall was intact; disruption of the muscle layer by enhanced tumor tissue was considered diagnostic of infiltration. In the present case, the CEUS images showed a loss of planes between the lesion and the bladder wall layers, with disappearance of the hyper-enhanced submucosal layer and hypo-enhanced muscle layer due to the presence of the hyper-enhanced tumor tissue. The disruption of the bladder wall muscular layer by enhancing tumor tissue confirms the infiltrative feature of the tumor [14].

CEUS with TICs could provide objectively and quantitatively parameters of the blood perfusion of the bladder tumor and reflect the angiogenesis of the tumor. Angiogenesis plays important role in the vascularization, growth, and metastasis of tumors. It is reported that a early wash-out enhancement pattern is common in arteriovenous fistulas as a result of angiogenesis, with a less distribution of tortuous vessels and interstitial oedema vessels related to a quick wash-out of contrast agent from the blood vessels [10]. In this case, the quantitative analysis showed the TIC characterized by high signal intensity, rapid wash-in, and early wash-out. This is similar to the results of Guo et al. [10], which displayed tumor with high-grade microvessel density, with high signal intensity and a fast wash-out. Therefore, CEUS, with TIC analysis, may be a useful non-invasive method in preoperatively evaluating the angiogenesis of bladder carcinoma.

CEUS provides useful information in diagnosing the bladder nodules, especially in the identification of clots and tumors, which could detect the vascularization of bladder cancer whereas bladder clots show no enhancement during all phases [12]. However, the presence of focal or nodular enhancement does not always lead to a definite diagnosis of bladder cancer. Thus, further studies are needed to prove the usefulness of CEUS in discriminating different bladder lesions such as adhered bladder lithiasis, bladder wall trabeculation, enlarged prostate or different pathologic type of bladder cancer.

However, CEUS has some limitations, similar to other ultrasound techniques. First, as with conventional sonography, obesity, calcifications, insufficient or excessive bladder distention and the lesion site can impair the quality of the bladder lesion image [7]. Second, CEUS is more dependent on operator practice and the experience of the physician. Third, it is difficult or impossible to obtain information on the extent of extravesical spread of widely infiltrating tumors or lymph node metastasis.

In summary, CEUS can well depict some common characteristics, which provide helpful clues in the diagnosis and detection of the depth of wall invasion of LCNEC in the urinary bladder. Furthermore, CEUS with TIC parameters could also be useful in the detection of microvasculature in LCNEC. We believe that CEUS may be an effective method for screening and diagnosing of LCNEC in the urinary bladder. Further studies involving more patients are mandatory to confirm these encouraging results.

## Data Availability

Not applicable.

## Abbreviations

LCNEC: Large cell neuroendocrine carcinoma
CEUS: Contrast-enhanced ultrasound
SMI: Superb microvascular imaging
TIC: Time-intensity curves
SCNEC: Small cell neuroendocrine carcinoma
MRI: Magnetic resonance imaging
CT: Computed tomography
